# Ag@Au core-shell dendrites: a stable, reusable and sensitive surface enhanced Raman scattering substrate

**DOI:** 10.1038/srep14502

**Published:** 2015-09-28

**Authors:** Hong Jun Yin, Zhao Yang Chen, Yong Mei Zhao, Ming Yang Lv, Chun An Shi, Zheng Long Wu, Xin Zhang, Luo Liu, Ming Li Wang, Hai Jun Xu

**Affiliations:** 1 Beijing Key Laboratory of Bioprocess, Beijing University of Chemical Technology, Beijing, 100029, China; 2 Engineering Research Center for Semiconductor Integrated Technology, Institute of Semiconductors, Chinese Academy of Sciences, Beijing, 100083, China; 3 Analytical and Testing Center, Beijing Normal University, Beijing 100875, China; 4 College of Sciences, Yanshan University, Qinhuangdao, 066004, China

## Abstract

Surface enhanced Raman scattering (SERS) substrate based on fabricated Ag@Au core-shell dendrite was achieved. Ag dendrites were grown on Si wafer by the hydrothermal corrosion method and Au nanofilm on the surface of Ag dendritic nanostructure was then fabricated by chemical reduction. With the help of sodium borohydride in water, Au surface absorbates such as thiophene, adenine, rhodamine, small anions (Br^–^ and I^–^), and a polymer (PVP, poly(N-vinylpyrrolidone)) can be completely and rapidly removed. After four repeatable experiments, the substrate SERS function did not decrease at all, indicating that the Ag@Au dendrite should be of great significance to SERS application because it can save much resource. Six-month-duration stability tests showed that the Ag@Au core-shell dendrite substrate is much more stable than the Ag dendrite substrates. We have also experimented on fast detection of Cd^2+^ at 10^−8^  M concentration by decorating single-stranded DNA containing adenine and guanine bases on the surface of this Ag@Au dendrite. Finite-difference time-domain simulations were carried out to investigate the influence of Au nanolayer on Ag dendrites, which showed that the local electric fields and enhancement factor are hardly affected when a 4 nm Au nanolayer is coated on Ag dendrite surface.

Surface enhanced Raman scattering (SERS) has been explored as an effective molecular imaging optical modality due to its inherent ability to generate enhanced Raman signal of analyte when it is in close proximity to nano-roughened noble metal surfaces like silver (Ag) or gold (Au)^1^,^2^,^3^. Recently, the SERS technique has been recognized as one of the most powerful and sensitive spectroscopic tools in the biological, clinical, chemical and environment fields because it can provide rich structural information in a non-destructive manner^2^,^4^,^5^,^6^,^7^. Many different Ag structures and substrates, which exhibit strong and tunable surface plasmon resonance (SPR) from the visible to the near-infrared spectral regimes^8^,^9^, have been proposed as effective enhancers of the weak basic Raman signals from the probe molecules. Compared to other structures, Ag dendrites possess many multi-level branching nanostructures, thus allowing a large specific surface area and the corresponding complex nanostructure may be more favorable to absorption of probe molecules^10^,^11^. Actually, the strong electromagnetic (EM) coupling can be formed in the space between two adjacent branches from the coupling of SPR. Thus a large amount of ‘hot spots’ would exist in the spaces at the end of branches or among adjacent Ag branches. These factors should favor Ag dendritic nanostructures to be used as high-active SERS substrates^2^,^8^.

Although Ag nanostructures possess high SERS properties in SERS applications, they have poor time-stability since their surface is highly sensitive to oxidation. To overcome this problem, many nanolayer shells on Ag cores (such as Ag/carbon, Ag/Au, and Ag/SiO_2_) were fabricated to improve the Ag nanostructure’s time-stability^12^,^13^,^14^. On the other hand, Au may be a promising SERS substrate material because 1) it has excellent stability, reusability, and biocompatibility, and 2) an Au nanolayer thinner than 5 nm can not influence the sensitivity of Ag-based SERS substrates^15^. Therefore, facile preparation of an Au nanolayer shell on Ag dendrites to form a time-stable and high sensitive SERS substrate can be highly desirable.

Additionally, most of SERS substrates are one-off, greatly limiting their SERS applications because of the waste of noble metals and high costs. Therefore, a recyclable SERS active substrate will save resources and provide access to better economic efficiency in addition to avoiding environment problems. On the other hand, the SERS application of Au nanostructures is currently restricted to the detection of organothiols (OTs) or thiolated biomolecules. Previous OT removal methods such as ultraviolet/ozone or ultraviolet photo-oxidation treatments are destructive methods and hinder the establishment of a quantitative relationship between substrates and analytes^16^,^17^. Recently, Yuan *et al.* showed that OTs on a planar Au film can be desorbed using sodium borohydride (NaBH_4_) in water^18^. Scott *et al.* reported that the thiolate in monolayer-protected Au clusters can be desorbed with a large excess of NaBH_4_ in tetrahydrofuran^19^. Both these results are consistent with the fact that borohydride can readily, rapidly and completely remove all the molecular adsorbates tested on the Au surfaces. These adsorbates include rhodamine 6 G (R6G), thiophene, adenine, small anions (I^–^ and Br^–^), polymer (PVP, poly(N-vinylpyrrolidone))^17^.Therefore the NaBH_4_ solution should have a good ability to clean many kinds of analytes such as R6G, Methyl orange (MO), p-Aminothiophenol(PATP) and Crystal violet (CV), etc. Accordingly, we can expect that it should be possible to achieve environment-friendly, durable and reusable Au-based SERS substrates with the help of NaBH_4_. In particular, the reusability of Au-based SERS substrates is environment as well as resource friendly.

The toxic heavy metal cadmium is a pollutant that threatens human health^20^. Cadmium ions (Cd^2+^) in food or water can cause chronic intoxication, decline of kidney function, rarefaction of bone and even cancer. A fast detection method for Cd^2+^ is therefore of importance. In this paper, a durable, reusable, highly stable and sensitive Ag@Au core-shell dendrite as active SERS substrate was prepared and used for quantitatively detecting Cd^2+^ through the chelation between the Cd^2+^ and thiolated single-stranded DNA (ssDNA)^21^,^22^. First, a simple and cost-effective method based on hydrothermal etching was used to prepare the desired Ag dendrite-based SERS substrate. The Ag dendrites were successfully grown on a Si wafer by hydrothermal corrosion. During the process, galvanic cell took place and Ag^+^ was reduced to Ag^23^. Next, an Au nanofilm was formed by chemical reduction on the surface of the Ag dendritic nanostructure. Recycling of the Ag@Au core-shell dendrite SERS substrate can be realized with the help of NaBH_4_ in water. Besides the mentioned outstanding properties, the substrates can also detect the R6G of concentration as low as 10^−8^ M. Finally, we use the Ag@Au core-shell dendrite SERS substrate to detect Cd^2+^ of concentration around 10^−8^ M with the help of ssDNA.

## Results and Discussion

**Figure 1:**
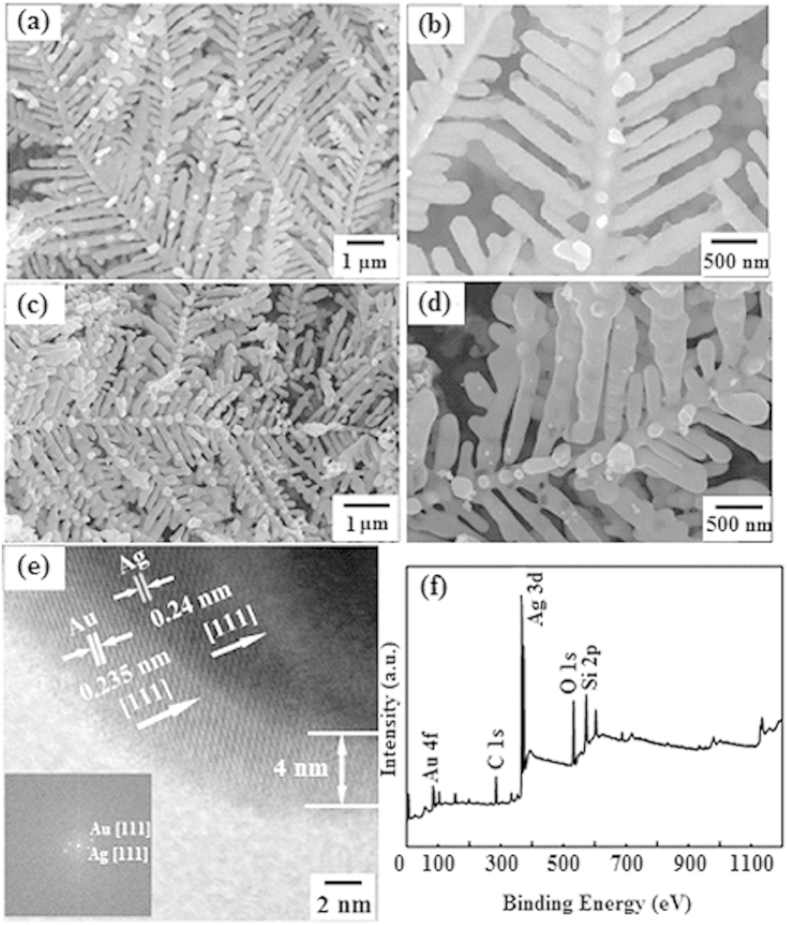
(**a**) Large-scale and (**b**) magnified FE-SEM images of Ag dendrites. After Ag dendrite coated with Au, the (**c**) lager-scale and (**d**) magnified FE-SEM images. (**e**) HRTEM image of the surface region of Au nanofilm covered Ag dendrite. (**f**) XPS spectra of Ag@Au core-shell dendrites.

Figure 1(a,b) show the field emission scanning electron microscopy (FE-SEM) images of the Ag dendritic nanostructures under different magnifications in the optimum etching condition which was 10 ml HF (10%) solution, 29 ml deionized water, and 1 ml H_2_O_2_ (30%) and 9 mg AgNO_3_, reacted at 50 ^o^C for 15 min. The same multi-level branching morphology and structure can be seen in Fig. 1(c,d) after an Au nanolayer shell was formed on the surface of Ag dendritic nanostructure. The size of the nanostructure is almost the same as that of the primordial Ag dendritic substrate. Sharp stems, symmetrical branches and leaves can be clearly observed. This Ag@Au core-shell dendrite can therefore be an ideal SERS substrate since it possesses a large amount of ‘hot spots’ in the spaces at the end of branches or among adjacent Ag branches. Actually, the EM and the chemical or charge transfer (CT) effects have been postulated for SERS enhancement: the former mainly due to the EM resonant excitation of localized SPR and the latter due to interaction among the organic molecules and their proximal metallic nanostructures^24^,^25^,^26^. It is widely believed that the long-range EM enhancement (of the order 10^4^–10^6^) plays a much greater role in SERS enhancement than the short-range CT enhancement (of the order 10^2^), particularly in most Ag/Au-based SERS systems^27^. Based on the mentioned morphological and structural analysis of the Ag@Au core-shell dendritic nanostructure, interpretations of the strong local EM effect have been given^28^,^29^,^30^. Choi *et al.* suggested that higher EM enhancements for metal nanoparticles can be achieved by reducing their gap distance to below 30 nm^31^. In this paper the estimated average diameters of Ag@Au trunks, branches and sub-branches were about 120, 80 and 40 nm, respectively, and the average spacing between the adjacent sub-branches was ~20 nm. Figure 1(e) shows the high-resolution transmission electron microscopy (HRTEM) image of an individual sub-branch. As can be seen, the thickness of the continuous nanolayer coated on the surface was approximately 4 nm and the lattice fringes of the Ag dendrite and Au nanolayer were about 0.240 and 0.235 nm, which correspond to the interplanar spacings of {111} of fcc Ag and Au crystals respectively. Furthermore, in the growth process of Ag dendrites on a Si wafer by hydrothermal corrosion, Ag nanoparticles adhere to form clusters, and this growth of these clusters is then controlled by the lowest surface energy, allowing the formation of dendritic Ag nanostructures. The Ag with different crystallographic planes features different surface energies. The general sequence 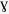
{111} < 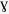
{100} < 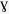
{110} holds for most fcc crystals. Therefore, one can conclude that the crystal preferentially grew along the {111} direction. In order to determine the phase of the crystalline zone, electron diffraction measurement with two-dimensional fast Fourier transform (FFT) mode was carried out. The result presented in the inset of Fig. 1(e) showed that the spots correspond to the diffraction from the (111) crystal plane of fcc Ag crystals. X-ray photoelectron spectroscopy (XPS) was used to examine the oxidation state for Au and Ag. The XPS image in Fig. 1(f) further confirms the formation of the substrate. In fact, the XPS peaks at 368.18 and 374.54 eV correspond to the 3d_5/2_ and 3d_3/2_ peaks, respectively, of metallic Ag, and the XPS peaks at 84.12 and 87.40 eV correspond to the 4f_7/2_ and 4f_5/2_ peaks, respectively, of metallic Au. These results demonstrate that in this case the Au and Ag are only in the zero-valent state. We can therefore conclude that the elemental Ag dendrites were coated by a continuous Au nanofilm.

**Figure 2:**
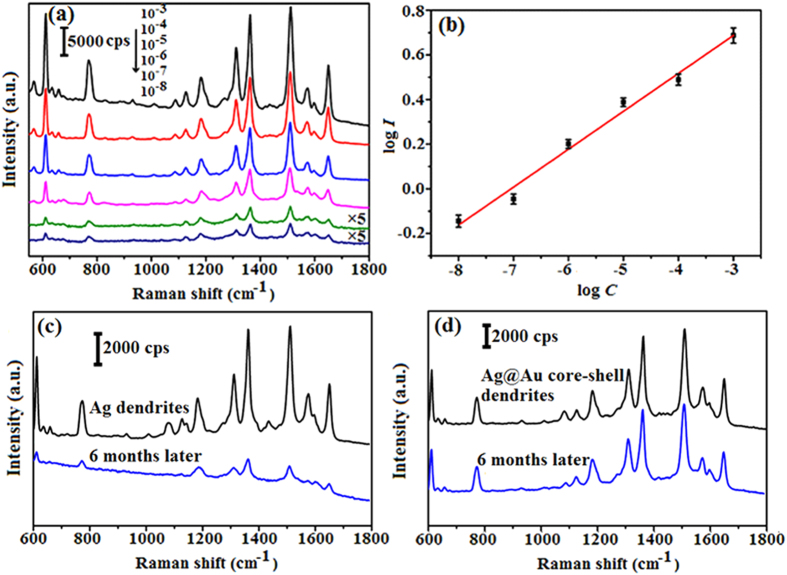
(**a**) SERS spectra of R6G obtained at different concentrations (from 10^−3^ to 10^−8^ M) with Ag@Au core-shell dendrites. (**b**) The linear relationship between log *I* of the band peaking at 1510 cm^−1^ and log *C*. (**c**,**d**) SERS spectra of 10^−5^ M R6G detected at Ag dendrites and Ag@Au core-shell dendrites respectively, here new-prepared substrates and substrates after six months were tested.

The 10^−3^ M R6G aqueous solution was prepared and the R6G solution of various concentrations such as 10^−4^, 10^−5^, 10^−6^, 10^−7^, and 10^−8^ M can be obtained by diluting it with deionized water. Figure 2(a) shows the SERS spectra of different-concentration R6G solution dispersed onto the Au-coated Ag dendrite substrates. Strong and medium-strong Raman bands from 10^−3^ to 10^−8^ M R6G solutions were observed. The SERS detection level of R6G for Ag@Au core-shell dendrites was as low as 10^−8^  M, which fully meets the requirements of single molecule detection^2^,^4^. Spectral characteristics of R6G are at about 610, 772, 1120, 1190, 1360, 1510, 1572 and 1649 cm^−1^, which can be attributed to the C-C-C ring in-plane, out-of-plane bending, C-H in-plane bending vibrations and C-C in-plane stretching vibrations^32^. Figure 2(b) gives the linear relationship between the logarithmic integrated intensity (log*I*) of the peak centered at 1510 cm^−1^ and the logarithmic concentration (log*C*) (here each datum indicates an average over 20 randomly selected positions). Clearly, the higher concentration of R6G leads to stronger Raman spectra with a linear relationship on a log-log plot, which allowed calibration of our substrate and determination of unknown concentrations of R6G solutions. Figure 2(c,d) show the stability evaluation of Ag dendrites and Ag@Au core-shell dendrites substrates, respectively. As shown in Fig. 2(c), the SERS performance of the Ag dendrites substrate without the Au coating deteriorates sharply six months later due to oxidation. Characteristic peaks of the R6G can be observed with difficulty and the enhancement effect of the Ag dendrites substrate is very weak. In contrast, Fig. 2(d) clearly shows that the characteristic peak intensities and positions of the Ag@Au core-shell dendrites substrate do not change over the six months, indicating that the Ag@Au core-shell dendrites substrate has very good time-stability.

Furthermore, we have compared the performance of our substrates with a conventional surface composed of aggregated Au colloids. Figure S1 gives the transmission electron microscopy (TEM) image of the Au colloidal suspension. The diameter of the Au nanoparticles in the Au colloidal suspension is almost 60 nm. Figure S2(a) presents the SERS spectra of different concentrations of R6G solution dispersed onto the aggregated Au colloids substrates. Clearly, the SERS detection level of R6G for the aggregated Au colloids substrate was almost 10^−7^ M, but for our Ag@Au core-shell dendrites substrate, the detection limit can be as low as 10^−8^ M. Both the detection limit and the signal intensity are different for the two cases (our substrate and the conventional aggregated Au colloids). Figure S2(b) shows the SERS spectra of R6G about 10^−5^ M on Ag@Au core-shell dendrites and aggregated Au colloids substrates. Obviously, the performance of our substrates is better than the conventional ones. On the other hand, we have also experimentally investigated the intensity-concentration profile for the other three described analytes (CV, MO and PATP) and presented the detailed results in Supporting Information (see Figs S3, S4 and S5). Apparently, the detection limits of the three analytes are 10^−8^, 10^−7^ and 10^−8^ M, respectively. Figures S3(b), S4(b) and S5(b) give the linear relationship between the logarithmic integrated intensity (log*I*) of the characteristic peak and the logarithmic concentration (log*C*) (here each datum represents an average over 20 randomly selected positions).

**Figure 3:**
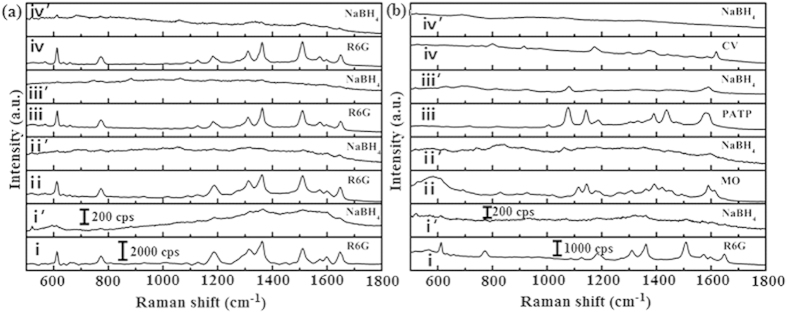
Recycle SERS behaviors of Ag@Au core-shell dendrites: (**a**) 10^−5^ M R6G with four cycles and (**b**) the alternating analysis of R6G, MO, PATP and CV with all 10^−5^ M concentration. Same labels of the odd and even curves in (**a**,**b**) respectively.

Next, we will examine the SERS recyclable applications of Ag@Au core-shell dendritic nanostructure substrate. By cleaning the substrate with NaBH_4_, we can reuse it many times. OTs and other analytes on gold nanoparticles can be rapidly and completely removed by NaBH_4_ treatment^17^,^19^,^33^. Two kinds of analytes, with (PATP) and without (R6G, MO and CV) OTs, were selected as probe molecules. Figure 3 shows the results for the analytes collected from the initial SERS detection and that after several washings. In all cases, the substrate was first immersed in a solution containing one corresponding analyte. It was then characterized by SERS and washed with a NaBH_4_ aqueous solution for 5 min. The substrate was further washed three times with water to remove residual ions and molecules and dried at 50 °C in vacuum. Here, all the Raman spectra were averaged over 10 different spatial positions. As shown in Fig. 3(a), curves I and I’ give spectra for 10^−5^ M R6G on the substrate before and after cleaning with NaBH_4_ solution. Comparing these two curves one can clearly find that the R6G signal disappeared completely after the first cleaning. Upon repeating this cycle three times (curves II ~ IV′ in Fig. 3(a)), the fingerprint peak of R6G on the cleaned substrate disappeared completely and the Raman spectra of the used substrate were similar to that of a new one. As shown in Fig. 3(b), this substrate was continuously dipped into solutions of R6G, MO, PATP and CV of the same concentration(10^−5^ M) and the detected SERS signals show that the Raman intensity was similar to that of the previously recycled R6G. This test of alternating SERS analytes shows that the Ag@Au core-shell dendritic nanostructure substrate is a feasible recyclable SERS substrate.

**Figure 4:**
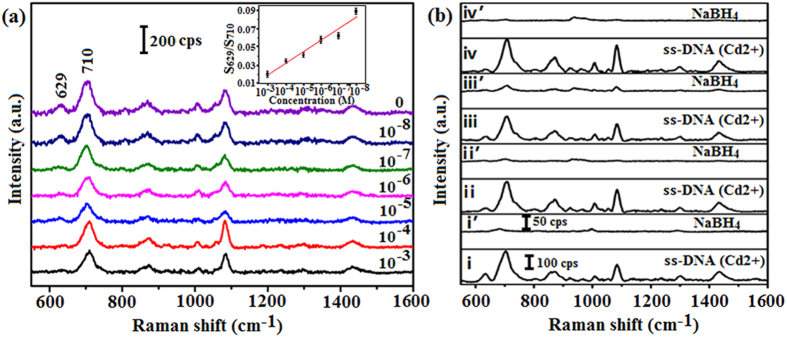
(**a**) Raman signals of Ag@Au core-shell dendrites decorated with ssDNA and different concentration of Cd^2+^ added on the surface of the substrate (from 10^−8^ to 10^−3^ M) compared with previous substrate without Cd^2+^. The inset shows the relationship between concentration of Cd^2+^ and the intensity ratio of the bands peaking at 710 and 629 cm^−1^. (**b**) Recycle SERS behaviors of Ag@Au core-shell dendrites after ssDNA decorated on the surface to detect Cd^2+^. Same labels of the odd and even curves in (**b**).

Heavy metal contamination may be very serious because of the use of heavy metal products, mining, emissions of exhaust, irrigation with sewage, etc. Among them, cadmium pollution is especially threatening for human health^20^. Here, we put forward a rapid detection method of Cd^2+^. Actually, it was reported that although all double-stranded DNA (dsDNA) samples yielded SERS spectra with good signal-to-noise ratios, none of the ssDNA oligomers studied yielded detectable SERS signals^34^. Meanwhile, the quality of the SERS spectrum of single-stranded Calf Thymus DNA was much better than that of the dsDNA^35^. More recently, SERS detection of both ss- and ds-DNA was reported, where the observed SERS features appeared to be sequence- and/or composition-dependent^36^. Before the Cd^2+^ is detected, it must be decorated by the thiolated ssDNA and the Raman spectra measured can be seen in Fig. 4(a) when the Cd^2+^ concentration was 0 M. We can get the characteristic peaks because of the existence of guanine, adenine, cytosine and thymine bases. Figure 4(a) suggests that, under our experimental conditions, the dominance of the adenine modes in the observed SERS spectra (~710 cm^−1^) is obvious and the intensity of this band is higher than the other characteristic peaks^22^. The SERS signal itself from the adenine bases appears to be more greatly enhanced than that of the other DNA bases. The observable SERS spectral signature from the guanine bases is the weak ~629 cm^−1^ peak, attributed to the ring breathing mode of guanine^22^,^34^,^37^,^38^. It is clear that the SERS spectral patterns of several thiolated ssDNA given in ref. 22 are all different from that presented in this paper. The existence of such differences can be considered as a normal phenomenon because the base sequence and base contents as well as excitation wavelength applied in these two cases are quite different. When excitation wavelength was different, the intensity and position of the characteristic peaks were also different and some feature peaks even disappeared. One can see that the characteristic peaks of the same assignment of analyte may have some shift reported by different cited papers because of various reasons and such small shift in a certain range can be considered to be reasonable^37^,^38^. As Cd^2+^ can generate chelation with the guanine bases and the intensity of the band peaking at ~629 cm^−1^ changes with the concentration of the Cd^2+^ dripped on the substrate. Meanwhile, the intensity of the adenine modes (~710 cm^−1^) throughout Fig. 4(a) is almost the same, regardless of the concentration of Cd^2+^. By choosing the band (whose intensity is almost a constant) peaking at ~710 cm^−1^as the interior label, we obtained the intensity ratio of the bands peaking at ~629 and ~710 cm^−1^. The relationship between concentration of Cd^2+^ and the integral area of discharge ratio of bands peaking at ~710 and ~629 cm^−1^ can be seen in the inset of Fig. 4(a). After we gained the quantitative relationship, the quantitative detection of Cd^2+^ was realized. Note that OTs and other analytes on Au surface can be rapidly and completely removed by NaBH_4_ treatment^17^,^18^,^19^. Figure 4(b) shows the SERS recyclable applications of Ag@Au core-shell dendritic nanostructure after ssDNA being decorated on the surface during detecting the Cd^2+^, where curves I and II give spectra for 10^−2^ M ssDNA with 10^−3^ M Cd^2+^ chelating with DNA on the substrate before and after cleaning with NaBH_4_ solution. Clearly, the DNA signal almost completely disappeared after the first cleaning. Upon repeating this cycle three times (curves II ~ IV′ in Fig. 4(b)), the fingerprint peak of DNA on the cleaned substrate disappeared completely. So, the verified result reveals that the recyclable application of the substrate is also tenable after it has been used in the Cd^2+^ detection.

**Figure 5:**
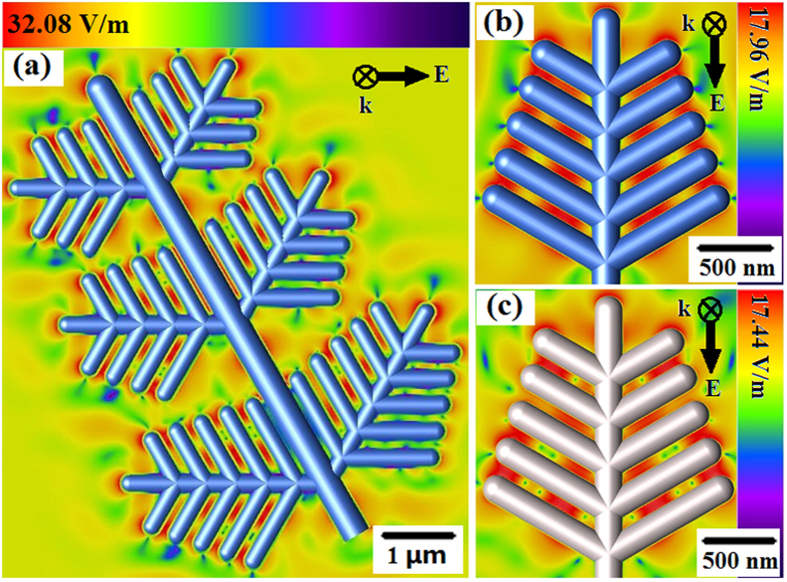
(**a**) Images of distribution of surface local electric field for Ag@Au dendritic nanostructure and image of distribution of surface local electric fields on second-level branch, (**b**) with and (**c**) without Au nanofilm.

Nanostructured Ag is known to yield the highest signal EFs in SERS^39^. Noting that the space between two adjacent branches became narrow after the Au nanolayer covered on the surface of Ag dendrites, we need to explore theoretically whether the signal EFs in SERS decreased after Au nanolayer formed. For this purpose, three-dimensional finite-difference time-domain (3D-FDTD) simulations were used to study the spatial distribution of the electric fields. Here, a commercial FDTD package (EMpro 2011 (64 bit)) was utilized to do the local EM field simulations. Figure 5(a) presents the profile of the local model Ag@Au dendritic branches, where the diameters of Ag@Au trunks, branches and subbranches were configured as ~120, 80, and 40 nm respectively and the spacing between adjacent subbranches was set as ~20 nm. The continuous wave laser with a wavelength of 633 nm propagating along *k* direction was input into the structure with its polarization direction perpendicular to *k*. Clearly, two types of ‘hot spots’ were formed: one presented among the neighboring Ag@Au branches and the other stemmed from the tip of each branch. The maximum calculated local electric field for the model Ag@Au dendritic nanostructures achieved ~32.08 Vm^−1^. So the design of complex multi-branch structures was strategic in enhancement of the electric field. Figure 5(b,c) display the spatial distributions of the calculated electric field intensities of the Ag sub-branches with and without 4 nm Au nanolayer. The calculated maximum local electric fields for the model Ag@Au and only Ag dendritic nanostructures reached ~17.96 and ~17.44 Vm^−1^, and the corresponding EFs are 1.04 × 10^5^ and 0.93 × 10^5^ respectively for the two cases. The simulation results show that with the ~4 nm Au layer on the Ag dendrite surface, the spatial distribution and the strength of the electric fields were very slightly changed. That is, the existence of ~4 nm Au nanolayer did not reduce the enhancement effect of the substrate, but improved the time-stability.

## Conclusion

In summary, Ag dendritic nanostructures were successfully grown on a Si wafer by using the method of hydrothermal corrosion and then Au nanolayer shell on the surface of the Ag dendrites was formed by chemical reduction method. The resulting substrate can detect R6G molecules with concentration as low as 10^−8^ M. Moreover, this substrate can be used as a recyclable SERS substrate with the help of sodium borohydride in water. The substrate can be applied not only to the same dye but also to different dyes with the ligand desorption method. After four repeatable experiments, the substrate can still be used without significant decrease in its function. The reusability of the substrate is of great significance to surface enhanced Raman scattering, which is valuable to the environment and can help to greatly save the resources. The experimental results of time-stability showed that after six months the Ag dendrite substrate cannot be used as a good SERS substrate because of oxidation to its surface, but the Ag@Au core-shell dendrite substrate can still give good SERS signals, confirming that the latter is highly stable in time. Meanwhile, rapid detection of Cd^2+^ is of extreme importance in treatment of cadmium pollution. In this paper, we have used our Ag@Au core-shell dendrite substrate to realize the rapid detection of Cd^2+^ with the help of thiolated ssDNA. The experimental results show that the substrate can also be used repeatedly after it has been used to test Cd^2+^ in practical application. Finally, the influence of Au nanolayer on the Ag dendrites has also been theoretically studied by means of the finite-difference time-domain simulations. The numerical results show that the electric fields as well as the enhancement factor are hardly influenced when a ~4 nm Au layer is coated on the Ag dendrite surface.

## Methods

### Preparation of Ag dendritic nanostructures

Four-inch n-type (111) oriented monocrystalline Si with a resistivity of 2–5 Ω·cm was used as the template to form Ag dendritic nanostructures. The Si wafer was ultrasonically cleaned by deionized water, acetone, and ethanol in sequence, each for 5 min. The cleaned wafer pieces were first immersed in 39 ml dilute HF (10%) solution for 15 min, then 1 ml H_2_O_2_ (30%) and 9 mg AgNO_3_ were mixed into the solution for another 15 min. Both the reaction temperatures were controlled at 50 ^o^C. During the hydrothermal etching process, Sireacted with H_2_O_2_ and then produced SiO_2_ and electron. Meanwhile, the Ag^+^ ions from AgNO_3_ obtained electron and produced metallic Ag dendrites. The growth of the Ag dendritic nanostructures is under non-equilibrium conditions and can be explained by the self-assembled localized microscopic electrochemical cell modeland the framework of a diffusion-limited aggregationprocess^40^. Ag nanoparticles adhere to form clusters, and the growth of these clusters is then governed by the lowest surface energy, allowing the formation of dendritic Ag nanostructures. The Ag with different crystallographic planes features different surface energies. The general sequence γ{111} < γ{100} < γ{110} holds for most face-centered cubic (fcc) crystals. So the growth direction of an Ag crystal is preferentially oriented parallel to the {111} direction. Additionally, the hydrothermal etching process to Si wafer provided reaction kinetics of Ag dendrite growth, the reaction might conduct rapidly where Si existed^40^. So Ag dendrites might grow evenly on the whole surface of Si template.

### Formation of Au nanolayer shell on the surface of Ag dendrites

HAuCl_4_ aqueous solution was prepared and the reaction was carried out at room temperature (20–25 °C). After Ag dendrite sample was immersed in 1 mM HAuCl_4_ solution for almost 60 s, Au nanolayer would be formed by the spontaneous reduction of Au^3+^ on the substrate through galvanic displacement. After that, the substrate was washed with deionized water. Thus the Ag@Au core-shell dendrite substrate was obtained.

### Raman detection and realization of reusability of the SERS substrate

The SERS measurement was conducted by LabRAM ARAMIS Raman system. The He-Ne (633 nm, 35 mW) laser was chosen as excitation source. Note that, among 532, 633 and 785 nm, 633 nm can be thought of as the most suitable excitation wavelength for obtaining good SERS spectra (the details can be found in Supporting Information, see Fig. S6). The D1 attenuator (tenth of the full power) was used and the diameter of laser spot was ~2 μm. The spectra were recorded with 1 s accumulation time and a twice cycle. All of the spectra in this work were collected under the conditions mentioned above. To reuse the SERS substrate, NaBH_4_ was selected to wash the adsorbates on the surface of Au nanofilm. Almost all the absorbates adsorbed on the substrate surface can be removed regardless of chemical adsorption (e.g., with OTs (PATP)) or physical adsorption (e.g., without OTs(R6G, MO and CV)). In all cases, the substrate was first immersed in a solution containing one corresponding analyte, then characterized by SERS and finally washed with 25 mM NaBH_4_ aqueous, that is to say, immersed the substrate in the 25 mM NaBH_4_ aqueous for 5 min. The substrate was then washed with water to remove residual ions and molecules and dried at 50 °C under vacuum. For Raman detection, R6G, MO, PATP and CV, each with concentration of 10^−5^ M, were prepared in advance.

### Detection of Cd^2+^ by decorating single-stranded DNA

SsDNA (5′-CCgATgTCgCAACAgTgAgCAgTCAC(Dual SH)-3′) was decorated on the surface of the Ag@Au core-shell dendrite substrate. The DNA buffer was prepared with phosphate solution and the final concentration of the DNA buffer was 10^−2^ M. Before the Cd^2+^ ion detection, SERS substrate prepared in advance must be immersed in the DNA buffer for 30 min. Because of the effect of –SH, DNA can connect on the surface of Ag@Au core-shell dendrites. Cd^2+^ ions can generate chelation with the guanine bases and therefore the intensities of some Raman bands would change. By choosing interior label with other Raman bands, the quantitative relationship can be obtained, which leaded to the quantitative detection of Cd^2+^ ions.

### FDTD simulations

The software we have used is EMpro 2011 (64 bit). In the FDTD simulations, we considered that Au and Ag abide by Debye-Drude Model. The values of parameters used for Ag are as follows: 1.15 × 10^7^  S/m (conductivity), 3.834 (infinite relative dielectric constant), −9530 (static relative dielectric constant), and 7.35 fs (relaxation time). The parameters used for Au are as follows: 1.525 × 10^7^  S/m (conductivity), 9.012 (infinite relative dielectric constant), −16000 (static relative dielectric constant), and 9.3 fs (relaxation time). The incident laser is of 632.8 nm wavelength and sine wave. The electric field intensity of the incident laser is 1 V/m. Computational grid size (in the target) is 6 nm and time step is about 0.0806613 fs. Detect convergence applied is −30 dB.

## Additional Information

**How to cite this article**: Jun Yin, H. *et al.* Ag@Au core-shell dendrites: a stable, reusable and sensitive surface enhanced Raman scattering substrate. *Sci. Rep.* **5**, 14502; doi: 10.1038/srep14502 (2015).

## Supplementary Material

Supplementary Information
