# Supplementary material for: Ag@Au core-shell dendrites: a stable, reusable and sensitive surface enhanced Raman scattering substrate
Source: Sci Rep. 2015 Sep 28;5:14502. doi: 10.1038/srep14502 (PMC4585979; doi:10.1038/srep14502)
Supplement: Supplementary Information [file srep14502-s1.doc]

**Supporting information**

**Ag@Au core-shell dendrites:** **a stable, reusable and sensitive surface enhanced Raman scattering substrate**

**Hong Jun Yin^1^, Zhao Yang Chen^1,*^, Yong Mei Zhao^2^, Ming Yang Lv^1^, Chun An Shi^1^, Zheng Long Wu^3^, Xin Zhang^1^, Luo Liu^1^, Ming Li Wang^4^, Hai Jun Xu^1,*^**

*^1^Beijing Key Laboratory of Bioprocess, Beijing University of Chemical Technology, Beijing, 100029, China*

*^2^Engineering Research Center for Semiconductor Integrated Technology, Institute of Semiconductors, Chinese Academy of Sciences, Beijing, 100083, China*

*^3^Analytical and Testing Center, Beijing Normal University, Beijing 100875, China*

*^4^College of Sciences, Yanshan University, Qinhuangdao, 066004, China*

* Corresponding authors:

Tel.: +86-10-64442357; Fax: +86-10-64435710

E-mail address: chenzy@mail.buct.edu.cn (Z.Y.Chen); hjxu@mail.buct.edu.cn (H. J. Xu)

**FIGURE CAPTIONS**

**FIGURES1** TEM of the Au colloidal suspension. The diameter of the Au nanoparticles in the Au colloidal suspension is almost 60 nm.

**FIGURES2**(a) SERS spectra of different-concentration R6G (10^-3^ to 10^-7^ M) detected on the aggregated Au colloids substrate; (b) SERS spectra of R6G with concentration of about 10^-5^ M on Ag@Au core-shell dendrites and aggregated Au colloids substrates respectively.

**FIGURES3** (a) SERS spectra of CV obtained at different concentrations (from 10^-3^ to 10^-8^ M) with Ag@Au core-shell dendrites. (b) The linear relationship between logI ofthe band peaking at 1620 cm^−1^ as a function of logC

**FIGURE S4** (a) SERS spectra of MO obtained at different concentrations (from 10^-3^ to 10^-7^ M) with Ag@Au core-shell dendrites. (b) The linear relationship between logI of the band peaking at 1170 cm^−1^ as a function of logC

**FIGURE S5** (a) SERS spectra of PATP obtained at different concentrations (from10^-3^ to 10^-8^ M) with Ag@Au core-shell dendrites. (b) The linear relationship between logI of the band peaking at 1075 cm^−1^ as a function of logC

**FIGURE S6** SERS spectra of R6G obtained at different excitation wavelengths (532 nm, 633 nm and 785 nm).

**FIGURE LIST**

**FIGURE S1**

**
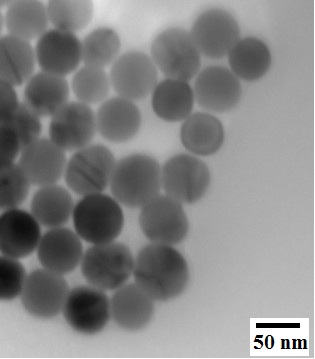
**

**FIGURE S2**

**
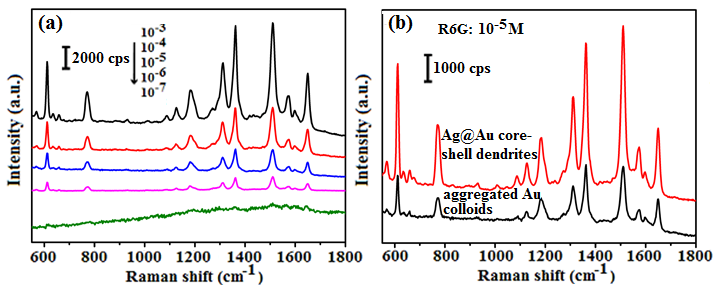
**

**FIGURE S3**

**
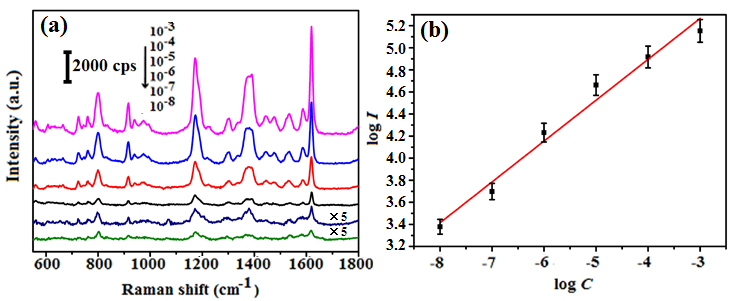
**

**FIGURE S4**


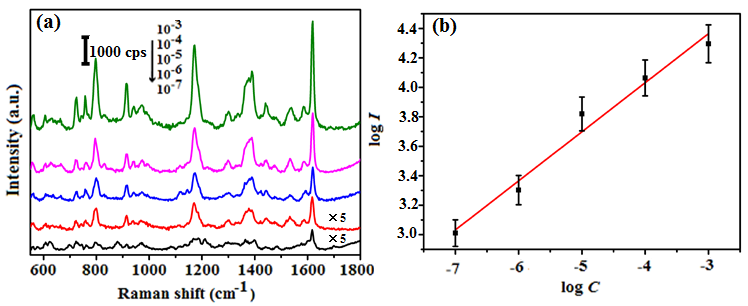


**FIGURE S5**


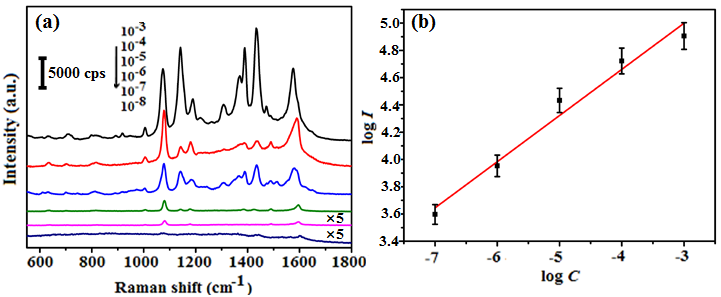


**FIGURE S6**

**
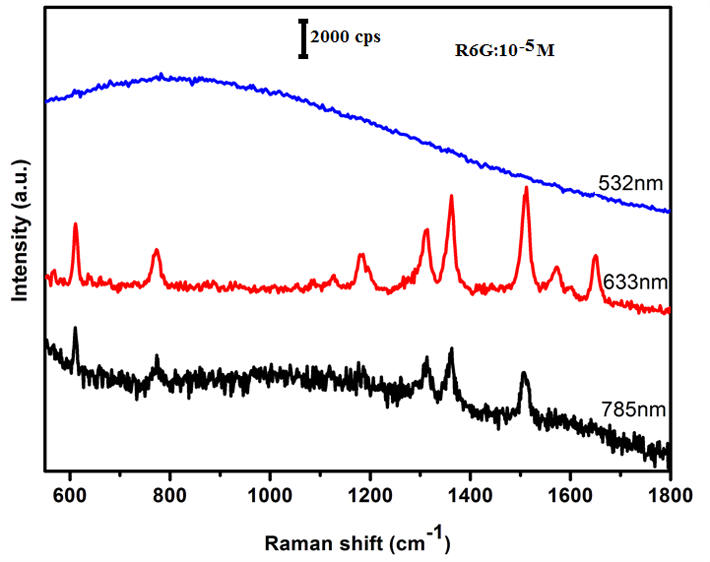
**
